# *Clostridium butyricum* Ameliorates Atherosclerosis by Regulating Host Linoleic Acid Metabolism

**DOI:** 10.3390/microorganisms13061220

**Published:** 2025-05-27

**Authors:** Chao Yin, Peizhi Fan, Xiangyu Mou, Wenjing Zhao

**Affiliations:** Shenzhen Key Laboratory for Systems Medicine for Inflammatory Diseases, School of Medicine, Shenzhen Campus of Sun Yat-Sen University, Shenzhen 518107, China; yinch23@mail2.sysu.edu.cn (C.Y.); fanpzh@mail2.sysu.edu.cn (P.F.)

**Keywords:** *Clostridium butyricum*, AS, linoleic acid, inflammation

## Abstract

Dysbiosis of the gut microbiota is strongly implicated in atherosclerosis (AS), thus prompting microbial modulation to be explored as a therapeutic strategy. However, limited evidence exists for probiotic interventions capable of alleviating AS. Here, we focused on *Clostridium butyricum* (*C. butyricum*; CB), a probiotic known for its production of short-chain fatty acids (SCFAs). We found that administration of *C. butyricum* to high-fat diet (HFD)-fed *Apoe* deficient (*Apoe^−/−^*) mice reduced plaque area by improving blood lipid profiles, decreasing macrophage infiltration in the aortic roots, and lowering the levels of circulating pro-inflammatory monocytes and macrophages. By non-targeted serum metabolomics analysis, *C. butyricum* treatment significantly reduced the levels of both linoleic acid and its downstream metabolites. Collectively, these findings establish *C. butyricum*-mediated amelioration of AS through modulation of linoleic acid metabolism.

## 1. Introduction

The gut microbiota serves as a central homeostatic regulator, orchestrating systemic health through dynamic crosstalk with host physiological networks [1]. Recent studies have indicated that the gut microbiota regulates host metabolic processes and immune homeostasis through specific microbial or shared host-microbiota metabolites, including SCFAs, secondary bile acids, and indole derivatives [2,3,4,5]. Emerging evidence has established gut microbial dysbiosis as a critical factor implicated in the pathogenesis of multiple systemic disorders, including cardiovascular disease (CVD), inflammatory bowel disease (IBD), and metabolic dysfunction-associated conditions [6,7,8,9,10,11]. Alterations in gut microbial composition, diminished biodiversity, and reduced species richness collectively drive the initiation and progression of these disorders [12,13]. Notably, Ambat et al. identified specific microbial deficiencies in IBD patients, demonstrating marked depletion of protective species including *Coprococcus comes*, *Butyricimonas paravirosa*, *Megasphaera indica* and *Agathobaculum butyriciproducens* [14]. Functional studies further revealed these bacteria ameliorate dextran sodium sulfate (DSS)-induced colitis by enhancing intestinal mucus barrier function. Similarly, microbial depletion profiles marked by reduced mucoprotective taxa have been observed in individuals with major CVD risk factors, particularly those presenting dyslipidemia, hypertension, or related metabolic disorders [15,16,17,18]. This consistent pattern of dysbiosis across various diseases suggests that gut microbiota acts as a system-wide regulator, where disrupted microbial consortia trigger multi-organ pathology.

AS, the primary pathological basis of CVD, is a chronic inflammatory disorder driven by dyslipidemia. This pathological process underlies atherosclerotic cardiovascular disease (ASCVD), characterized by the buildup of fats, cholesterol, and other substances in arterial walls, resulting in a progressive constriction and stiffening of blood vessels. Studies have shown that alterations in gut microbiota composition, characterized by shifts in microbial diversity and the abundance of specific genera, have been linked to the progression of AS. Typically, Jie et al. demonstrated a significant depletion of *Bacteroides* and *Prevotella* genera in ASCVD accompanied by increased *Streptococcus* and *Escherichia* within gut microbiota [19]. Moreover, gut microbiota-derived tryptophan metabolites have been demonstrated to exert a pivotal role in the amelioration of ASCVD by dampening inflammation and the generation of reactive oxygen species (ROS) within endothelial cells [3,20]. Despite these insights, the clear causal relationships between the gut microbiota and AS progression remains poorly understood.

*C. butyricum*, an obligate anaerobe and a Gram-positive rod-shaped bacterium, is a commensal microorganism commonly found in the intestinal tracts of both humans and various animal species. *C. butyricum* has been demonstrated to restore intestinal barrier integrity through the fermentation of dietary fiber into SCFAs. Significantly, this probiotic has also been found to alleviate vascular inflammation and regulate the systemic balance between T helper cell 17 (Th17) and regulatory cells (Tregs) balance [21,22]. Therefore, we proposed that *C. butyricum* has the potential to inhibit the progression of AS. In this study, we revealed that *C. butyricum* colonization exhibits an atheroprotective effects in HFD-fed *Apoe^−/−^* mice. Furthermore, we demonstrated that *C. butyricum* suppressed AS progression in HFD-fed *Apoe^−/−^* mice through modulation of host linoleic acid metabolism, characterized by reduced body weight, decreased plaque area and macrophage infiltration in the vascular wall, ameliorated dyslipidemia, and suppressed systemic inflammation. Collectively, our findings reveal a crucial gut microbiota-host metabolic crosstalk, offering a microbiota-targeted therapeutic strategy for ASCVD management.

## 2. Materials and Methods

### 2.1. Mice

All experiments involving animals were performed in compliance with protocols authorized by the Institutional Animal Care and Use Committee (IACUC) at Sun Yat-sen University (Approval ID: SYSU-IACUC-2024-000526). Male C57BL/6 *Apoe*-deficient (*Apoe^−/−^*) mice were obtained from Gem Pharmatech (Nanjing, China) and maintained in strict specific pathogen-free facilities under standardized environmental conditions with 12 h light and 12 h dark phases. The mice were assigned to various experimental groups at random, and all mice were included in the analysis without exclusions. The mice were administered a high-fat diet (HFD; Synergy Bio., Nanjing, China), containing 20% protein, 21% fat, and 50% carbohydrate, for 12 weeks [23,24]. To evaluate the anti-atherosclerotic effects of *C. butyricum*, *Apoe^−/−^* mice were administered 100 μL of either PBS (vehicle control; Sangon Biotech, Shanghai, China) or *C. butyricum* suspension (10^9^ CFU/mouse) via oral gavage three times weekly for 12 weeks.

For biochemical profiling, plasma levels of triglycerides (TG), total cholesterol (TC), low-density lipoprotein cholesterol (LDL-C), and high-density lipoprotein cholesterol (HDL-C) were quantitatively determined using standardized assays with commercially available kits (Rayto, Shenzhen, China) in strict accordance with the manufacturer’s protocols.

### 2.2. Bacterial Preparation

The *Clostridium butyricum* CICC 10390 was purchased from China Center of Industrial Culture Collection (CICC, Beijing, China). *C. butyricum* was cultured in TSB medium (17 g/L tryptone, 3 g/L soya peptone, 5 g/L NaCl, 2.5 g/L K_2_HPO_4_, 2.5 g/L glucose; Hopebio, Qingdao, China) at 37 °C under anaerobic conditions with 85% N_2_, 10% H_2_, and 5% CO_2_ [25]. For *C. butyricum* treatment in *Apoe^−/−^* mice, bacterial pellets were harvested and resuspended in anaerobic PBS. Colony-forming units per mL (CFU/mL) was determined by performing plate counts on TSB agar. The *C. butyricum* suspension was adjusted to 1 × 10^9^ CFU per 0.1 mL and administered to mice [26].

### 2.3. Fecal DNA Extraction and qPCR Analysis

Fecal DNA extraction was performed using the TIANamp Stool DNA Kit (Tiangen, Beijing, China) according to the manufacturer’s protocol. In brief, approximately 50 mg fecal samples were subjected to sequential lysis, purification, and elution steps, with a final elution volume of 50 μL [27]. Quantitative PCR analysis for *C. butyricum* was performed using the 2 × SYBR Green qPCR Master Mix kit (Selleck Chemicals, Houston, TX, USA) on a QuantStudio 7 Pro Real-Time PCR system (Thermo Fisher Scientific, Waltham, MA, USA). The specific primers targeting *C. butyricum* were employed for amplification [28]. Primers sequences are detailed in Table S3.

### 2.4. Assessment of Atherosclerotic Lesions in the Whole Aorta and Aortic Root

Heart and whole aorta samples were surgically isolated, followed by immediate fixation in 4% paraformaldehyde (PFA; Biosharp, Hefei, China) for 24 h at 4 °C. Following careful dissection of residual perivascular adipose tissue under a stereomicroscope (Olympus, Tokyo, Japan), aortic samples were dehydrated in 20% sucrose solution at 4 °C for 24 h. To prepare frozen sections of the aortic root, heart tissues were embedded in optimal cutting temperature (OCT) compound. Both whole aorta specimens and the corresponding frozen sections were stained with Oil Red O solution (Sigma-Aldrich, St. Louis, MO, USA), followed by a quantitative analysis of the lesion areas using Image J software v1.53m.

### 2.5. Sample Preparation and Untargeted Metabolomics Analysis

Sample preparation was performed following an adapted protocol based on previously established methods [29]. Serum samples (50 μL) were combined with 300 μL of ice-cold extraction solvent (acetonitrile:methanol, 1:4 *v*/*v*) containing internal standards in 2 mL microcentrifuge tubes. After vortex mixing for 3 min, phase separation was achieved by centrifugation at 12,000 rpm for 10 min at 4 °C. Subsequently, 200 μL of the supernatant was incubated at −20 °C for 30 min, followed by a secondary centrifugation at 12,000 rpm for 3 min at 4 °C. Finally, 180 μL of the clarified supernatant was used for liquid chromatography-tandem mass spectrometry (LC-MS) analysis. Quality control (QC) samples were generated by equal-volume pooling of all experimental supernatants. The Waters ACQUITY UPLC HSS T3 C18 (1.8 µm, 2.1 mm × 100 mm) was used to separate the extracts. Chromatographic separation was performed on a column maintained at 40 °C with mobile phases consisting of (A) 0.1% formic acid in water and (B) 0.1% formic acid in acetonitrile, delivered at 400 μL/min. A 2 μL injection volume was employed with the following gradient program: 95% A (0–11 min), linearly decreased to 10% A (11–12 min), maintained for 2 min (12–14 min), followed by re-equilibration to initial conditions. Multivariate pattern recognition was implemented through principal component analysis (PCA) and partial least squares-discriminant analysis (PLS-DA) to characterize metabolic profiles distinguishing *C. butyricum*-treated groups from PBS controls. Data analysis was conducted using the R package v1.20.0. Annotated metabolites meeting dual criteria of variable importance in projection (VIP) scores >1.0 and *p* < 0.05 (two-tailed Student’s *t*-test) were designated as significantly altered compounds. Metabolite annotation was conducted via mzCloud (https://www.mzcloud.org/ accessed on 20 January 2025), HMDB (http://www.hmdb.ca accessed on 20 January 2025), and KEGG databases (http://www.genome.jp/kegg/ accessed on 20 January 2025) following previously established protocols. Pathway enrichment analysis of differential metabolites was conducted through KEGG ontology mapping. KEGG database was employed for enrichment analysis of significantly altered metabolites.

### 2.6. Immunofluorescence Analysis

Aortic root cryosections were fixed in 4% PFA for 15 min, followed by three washes in PBS, each lasting 5 min. Antigen retrieval was performed by heating sections in Tris-EDTA buffer (pH 9.0) at 95 °C for 20 min using a water bath, then equilibrated to room temperature for 30 min. Following three additional washes with PBS, nonspecific binding sites were blocked with 10% normal donkey serum (Solarbio, Beijing, China) for 30 min at room temperature. Sections were then incubated overnight at 4 °C with primary antibodies against CD68 (Servicebio, Wuhan, China), followed by 1 h incubation with Alexa Fluor 488-conjugated secondary antibodies (Servicebio, China) at room temperature. Nuclei were counterstained with DAPI-containing Fluoromount-G mounting medium. Fluorescent images were acquired using a fluorescence microscope (Nikon, Tokyo, Japan), with intensity quantification performed in ImageJ software v1.53m.

### 2.7. Flow Cytometric Analysis

Peripheral blood nucleated cells were isolated using 1× RBC Lysis Buffer (BioLegend, San Diego, CA, USA) for erythrocyte lysis, followed by washing with PBS. The cells were then immunolabeled in ice-cold PBS containing 0.2% BSA for 20 min with fluorochrome-conjugated monoclonal antibodies. Specifically, CD45-FITC (BioLegend, USA) was used for leukocyte identification; CD45-FITC, CD11b-PE (Tonbo Biosciences, San Diego, CA, USA), and Ly6C-PE-Cy7 (BioLegend, USA) were employed for monocyte subtyping; and for macrophage polarization analysis, a combination of CD45-FITC, CD11b-PE, F4/80-PE-Cy5 (Tonbo Biosciences, USA), CD86-APC-Cy7 (BioLegend, USA), and CD206-APC (BioLegend, USA) was utilized. Cellular acquisition was performed using a Novocyte D2060R cytometer (Agilent, Santa Clara, CA, USA), and subsequent data analysis was conducted in NovoExpress software v1.6.2 through sequential gating hierarchies.

### 2.8. Data Statistical Analysis

Data are presented as mean ± SEM. Cohen’s d was calculated to quantify the size effect difference between the two groups [30]. Between-group comparisons were performed using either unpaired two-tailed Student’s *t*-tests or two-way ANOVA with Tukey’s post hoc test in GraphPad Prism v9.0 (GraphPad Software), with statistical significance defined as *p* < 0.05.

## 3. Results

### 3.1. C. butyricum Inhibits Atherosclerosis in HFD-Fed Apoe^−/−^ Mice

**Figure 1:**
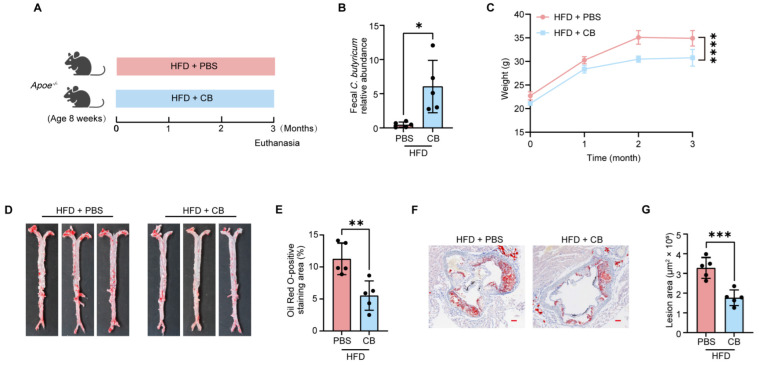
*C. butyricum* colonization attenuates HFD-induced body weight gain and atherosclerotic plaque formation in *Apoe^−/−^* mice. (**A**) Experimental design of the HFD-induced AS mouse model. Each group consisted of five mice. (**B**) The relative abundance of CB in fecal samples was quantified by qPCR with species-specific primers. (**C**) Body weight during *C. butyricum* treatment. (**D**,**E**) Representative whole aorta images stained with oil red O (**D**), along with the quantification of the lesion area (**E**). Representative whole aorta images stained with oil red O (**F**), along with the quantification of the lesion area (**G**). Data are presented as the mean ± SEM ((**B**,**C**,**E**,**G**); n = 5). Two-tailed Student’s *t* test (**B**,**E**,**G**) or two-way ANOVA with Turkey’s post hoc test (**C**) was used to determine *p*-values: * *p* < 0.05; ** *p* < 0.01; *** *p* < 0.001; **** *p* < 0.0001.

To explore the effect of *C. butyricum* on AS, *Apoe^−/−^* mice maintained on a high-fat diet (HFD) were administered either *C. butyricum* (CB) or PBS for 12 weeks (Figure 1A; effect size: 2.05, 95% confidence interval (CI): 0.52–3.57, *p* = 0.012). Successful colonization of *C. butyricum* was confirmed in the mice (Figure 1B). By the 2-month time point, *C. butyricum* treatment significantly attenuated HFD-induced body weight gain in mice (Figure 1C; effect size: 2.40, 95% CI: 0.49–4.31, *p* < 0.0001). Atherosclerotic lesion burden was evaluated by Oil Red O staining-based quantification of lipid deposition analyzed in both aortic root cross-sections and the entire aortic tree. Similarly, compared with PBS-treated mice, *C. butyricum*-treated group showed significant 52% and 46% reductions in entire aortic (Figure 1D,E; effect size: 2.41, 95% CI: 0.78–4.04, *p* = 0.0052) and aortic root lesion areas (Figure 1F,G; effect size: 2.55, 95% CI: 0.81–4.29, *p* = 0.0009), respectively. These data indicate that *C. butyricum* attenuates HFD-induced AS in *Apoe^−/−^* mice, evidenced by decreased body weight and atherosclerotic lesion.

### 3.2. Impact of C. butyricum Administration on the Serum Metabolic Profiles in HFD-Fed Apoe^−/−^ Mice

**Figure 2:**
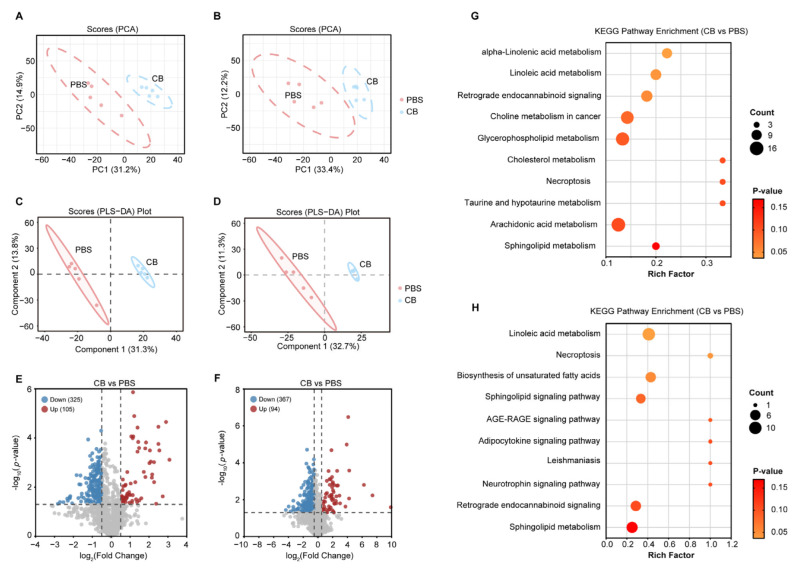
*C. butyricum* modulates serum metabolite profiles in HFD-fed *Apoe^−/−^* mice. (**A**,**B**) PCA in the negative (left) and positive (right) ionization modes (n = 5). (**C**,**D**) PLS-DA analysis in the negative (left) and positive (right) ionization modes (n = 5). (**E**,**F**) Volcano plots of differential serum metabolites between PBS and *C. butyricum*-treated groups in negative (left) and positive (right) ionization modes. (**G**,**H**) The KEGG pathway analysis of differential metabolites between PBS and *C. butyricum*-treated groups in negative (left) and positive (right) ionization modes.

To investigate the mechanism underlying the anti-atherosclerotic effects of *C. butyricum*, we performed LC-MS/MS analysis in dual ionization modes (negative/positive) to compare non-targeted serum metabolic profiles between the PBS- and *C. butyricum*-treated groups. Both the principal component analysis (PCA) and the partial least squares discrimination analysis (PLS-DA) revealed strikingly distinct metabolite profiles between the two groups (Figure 2A–D). Compared to the PBS-treated group, *C. butyricum* treatment significantly altered metabolite profiles, showing 325 downregulated and 105 upregulated metabolites in negative ionization mode (Figure 2E), along with 367 downregulated and 94 upregulated metabolites in positive ionization mode (Figure 2F).

To characterize the core metabolic networks modulated by *C. butyricum*, we systematically categorized altered pathways (Figure S1). Pathway analysis highlighted significant enrichment in polyunsaturated fatty acid (PUFA) metabolism, including linoleic acid metabolism, arachidonic acid metabolism, biosynthesis of unsaturated fatty acids, and α-linolenic acid metabolism. Notably, Kyoto Encyclopedia of Genes and Genomes (KEGG) pathway analysis revealed linoleic acid metabolism as the most prominent pathway identified in both ionization modes (Figure 2G,H and Tables S1 and S2). These data indicate that *C. butyricum* modulates the serum metabolic profile of HFD-fed *Apoe^−/−^* mice.

### 3.3. C. butyricum Modulates Host Linoleic Acid Metabolism

**Figure 3:**
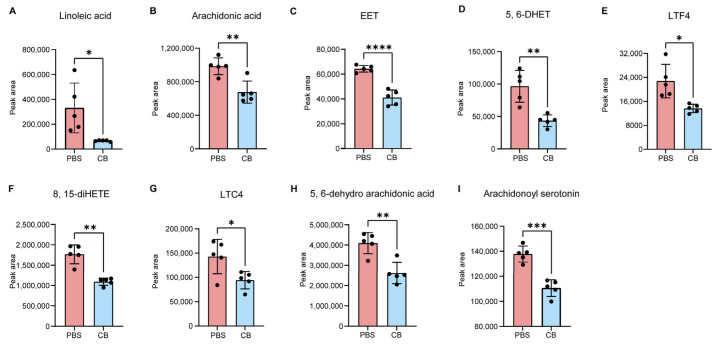
Comparison of the levels of linoleic acid and its derivatives after *C. butyricum* treatment. (**A**) Linoleic acid. (**B**) Arachidonic acid. (**C**,**D**) CYP450 pathway metabolites. (**E**,**F**) LOX pathway metabolites. (**H**,**I**) Arachidonic acid derivatives. Data are presented as the mean ± SEM ((**A**–**I**); n = 5). Two-tailed Student’s *t*-test was used to determine *p*-values (**A**–**I**): * *p* < 0.05; ** *p* < 0.01; *** *p* < 0.001; **** *p* < 0.0001.

Linoleic acid is converted into arachidonic acid (ARA) through a series of desaturation and elongation reactions catalyzed by specific enzymes [31]. The arachidonic acid is then metabolized via three distinct pathways: cyclooxygenase (COX), lipoxygenase (LOX), and cytochrome P450 (CYP450), generating bioactive derivatives [32,33]. To determine the effect of *C. butyricum* on linoleic acid metabolism, we further analyzed the contents of linoleic acid and its downstream metabolites based on the non-targeted metabolomics. We observed that the levels of linoleic acid (Effect size: 2.50, 95% CI: 0.76–4.24, *p* = 0.0185) and ARA (Effect size: 2.65, 95% CI: 0.95–4.35, *p* = 0.0031) were decreased in *C. butyricum*-treated group, compared with PBS-treated mice (Figure 3A,B). As CYP450-mediated metabolites of ARA [34,35,36], both epoxyeicosatrienoic acid (EET; effect size: 2.08, 95% CI: 0.58–3.58, *p* < 0.0001) and 5, 6-dihydroxyeicosatrienoic acid (5, 6-DHET; effect size: 1.90, 95% CI: 0.47–3.33, *p* = 0.0019) were significant down-regulated in HFD-fed *Apoe^−/−^* mice following *C. butyricum* administration (Figure 3C,D). Similarly, the LOX pathway was also remarkably inhibited in the non-targeted metabolomics analysis. The levels of leukotriene F4 (LTF4; effect size: 1.98, 95% CI: 0.54–3.42, *p* = 0.0075) [33], leukotriene C4 (LTC4; effect size: 1.40, 95% CI: 0.22–2.58, *p* = 0.026) [33] and 8,15-dihydroxyeicosatetraenoate (8,15-diHETE; effect size: 1.93, 95% CI: 0.51–3.35, *p* = 0.0003) [37] in *C. butyricum*-treated group were lower than in the PBS controls (Figure 3E–G). Moreover, additional ARA-derived metabolites including 5, 6-dehydro arachidonic acid (effect size: 2.80, 95% CI: 1.05–4.55, *p* = 0.0022) [38] and arachidonoyl serotonin (effect size: 4.2, 95% CI: 1.98–6.42, *p* = 0.0002) [39] exhibited reduced levels in response to *C. butyricum* treatment (Figure 3H,I). These data highlight the capacity of *C. butyricum* to alter linoleic acid metabolic processes.

### 3.4. C. butyricum Reduces Circulating Pro-Inflammatory Monocytes by Improving HFD-Induced Dyslipidemia in Apoe^−/−^ Mice

**Figure 4:**
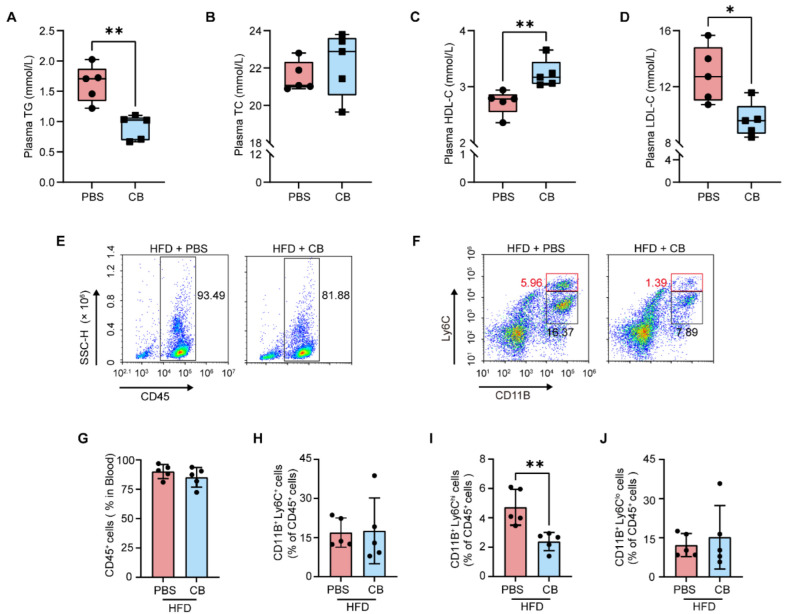
*C. butyricum* attenuates HFD-induced dyslipidemia and suppresses pro-inflammatory monocytes in *Apoe^−/−^* mice. (**A**–**D**) Plasma concentrations of triglycerides (TG; (**A**)), total cholesterol (TC; (**B**)), high-density lipoprotein cholesterol (HDL-C; (**C**)), and low-density lipoprotein cholesterol (LDL-C; (**D**)) were assessed. (**E**,**G**) Representative flow cytometer profiles (**E**) and quantification (**G**) of CD45^+^ leukocytes in peripheral blood samples. (**F**,**H**–**J**) Representative flow cytometer profiles (**F**) and quantification (**H**–**J**) of total monocytes (CD11B^+^ Ly6C^+^), CD11B^+^ Ly6C^hi^ monocytes, and CD11B^+^ Ly6C^lo^ monocytes in peripheral blood samples. Data are presented as the mean ± SEM ((**A**–**D**,**G**–**J**); n = 5). Two-tailed Student’s *t*-test was used to determine *p*-values (**A**–**D**,**G**–**J**): * *p* < 0.05; ** *p* < 0.01.

Dyslipidemia has been established as a pivotal driver of AS [40]. Notably, a recent study has revealed that wild-type mice maintained on a HFD exhibited significant dyslipidemia, along with elevated serum levels of linoleic acid and ARA, suggesting a potential link between altered linoleic acid metabolism and dyslipidemia [41]. To investigate this association, we conducted a comprehensive analysis of plasma lipid profiles. Compared to the PBS-treated group, *C. butyricum* administration significantly reduced triglyceride (TG; Figure 4A; effect size: 2.78, 95% CI: 0.69–4.89, *p* = 0.0023) and low-density lipoprotein cholesterol (LDL-C; Figure 4D; effect size: 1.28, 95% CI: −0.03–2.59, *p* = 0.0147) levels, while concurrently increasing high-density lipoprotein cholesterol (HDL-C; Figure 4C; effect size: 2.18, 95% CI: 0.34–4.03, *p* = 0.0087) concentrations, but had no significant effect on total cholesterol (TC; Figure 4B; effect size: −0.52, 95% CI: −2.10–1.05, *p* = 0.4279) levels. Given that plasma LDL-C levels showed a positive correlation with circulating pro-inflammatory monocytes [42,43,44], we conducted flow cytometric analysis to systematically quantify the distribution of leukocyte populations and monocyte subsets. We found that *C. butyricum* treatment significantly reduced pro-inflammatory monocytes (CD11B^+^ Ly6C^hi^; Figure 4F,I; effect size: 2.40, 95% CI: 0.49–4.31, *p* = 0.0052), while showing no notable effects on leukocyte populations (CD45^+^; Figure 4E,G; effect size: 0.68, 95% CI: −0.60–1.95, *p* = 0.3168), total monocyte numbers (CD11B^+^ Ly6C^+^; Figure 4F,H; effect size: 0.07, 95% CI: −1.17–1.31, *p* = 0.9131), or CD11B^+^ Ly6C^lo^ monocytes (Figure 4F,J; effect size: 0.33, 95% CI: −1.14–1.80, *p* = 0.6158). These data demonstrate that *C. butyricum* reduced circulating pro-inflammatory CD11B^+^ Ly6C^hi^ monocytes through improving dyslipidemia, with this effect mediated through its modulation of linoleic acid metabolic homeostasis.

### 3.5. C. butyricum Attenuates Macrophage Infiltration in the Aortic Root and Promotes M2 Macrophage Polarization

Linoleic acid, an oxidation-susceptible PUFA [45], undergoes peroxidation to produce bioactive metabolites that directly stimulate ROS and upregulate adhesion molecules within arterial walls [46]. These effects enhance endothelial permeability, promoting LDL deposition in the arterial intima, where oxidative modification yields oxidized LDL (ox-LDL) [46,47,48]. Intimal macrophages express scavenger receptors that mediate ox-LDL uptake, ultimately driving foam cell formation and atherosclerotic plaque development [49].

**Figure 5:**
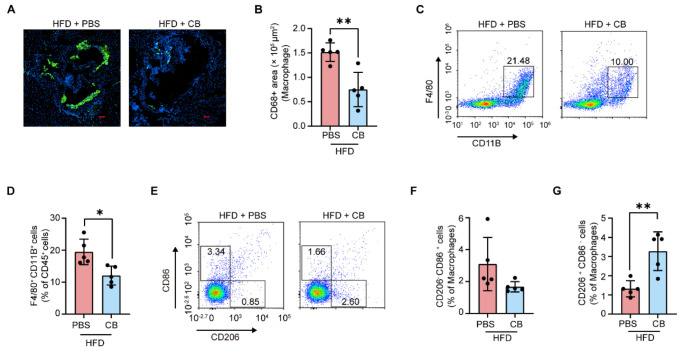
*C. butyricum* attenuates macrophage infiltration in the aortic root and promotes M2 macrophage polarization. (**A**,**B**) Immunofluorescence staining of CD68^+^ macrophages in the aortic roots. Representative images and quantification of CD68^+^ area. Scale bar: 100 μm. (**C**,**D**) Representative flow cytometer profiles (**C**) and quantification (**D**) of macrophages (F4/80^+^ CD11B^+^) in peripheral blood samples. (**E**–**G**) Representative flow cytometer profiles (**E**) and quantification (**F**,**G**) of M1 macrophages (CD206^-^ CD86^+^) and M2 macrophages (CD206^+^ CD86^-^) in peripheral blood samples. Data are presented as the mean ± SEM ((**B**,**D**,**F**,**G**); n = 5). Two-tailed Student’s *t*-test was used to determine *p*-values (**B**,**D**,**F**,**G**): * *p* < 0.05; ** *p* < 0.01.

To further determine the linoleic acid-dependent anti-atherosclerotic effects of *C. butyricum*, macrophage content in aortic roots was quantified in HFD-fed *Apoe^−/−^* mice after 12 weeks of oral gavage with PBS or *C. butyricum*. We observed that macrophage content was significantly reduced in *C. butyricum* -treated group (Figure 5A,B; effect size: 2.71, 95% CI: 0.57–4.85, *p* = 0.0026). Additionally, Valencia et al. identified that NLRP3 inflammasome in macrophages was activated by 12,13-dihydroxy-9z-octadecenoic acid (12,13-DiHOME), a CP450-mediated dihydroxy metabolite derived from linoleic acid [50]. We therefore hypothesized that linoleic acid might affect macrophage polarization dynamics, and accordingly conducted systematic profiling of circulating macrophage subsets in murine peripheral blood. Compared with PBS-treated group, the *C. butyricum*-treated group significantly increased M2 macrophage abundance (CD206^+^CD86^-^; Figure 5E,G; effect size: 2.54, 95% CI: 0.47–4.61, *p* = 0.0039), with a concomitant downward trend in CD11B^+^ F4/80^+^ macrophage levels (Figure 5C,D; effect size: 2.12, 95% CI: 0.30–3.94, *p* = 0.0102), whereas no significant alteration was observed in M1 macrophage populations (CD206^-^CD86^+^; Figure 5E,F; effect size: 1.18, 95% CI: −0.43–2.79, *p* = 0.0991). These findings demonstrate that *C. butyricum* suppresses macrophage accumulation within the aortic root while promoting macrophage polarization toward the M2 phenotype through modulation of linoleic acid metabolism.

## 4. Discussion

The gut microbiota has emerged as a pivotal player in the AS progression, yet the mechanisms underlying microbial modulation of host lipid metabolism and immune dynamics remain incompletely defined. This study elucidates a novel mechanism by which *C. butyricum* attenuates AS in HFD-fed *Apoe^−/−^* mice through targeted modulation of host linoleic acid metabolism. *C. butyricum* administration significantly reduced atherosclerotic plaque burden, improved dyslipidemia, and suppressed systemic inflammation, as evidenced by decreased circulating pro-inflammatory monocytes and aortic macrophage infiltration, alongside elevated circulating M2 macrophage populations. Non-targeted metabolomics revealed that *C. butyricum* treatment downregulated linoleic acid and its downstream metabolites, including ARA and its CYP450 and LOX-derived bioactive derivatives. Our data suggest that *C. butyricum* could serve as a therapeutic agent that interrupts linoleic acid-driven inflammatory cascades, thereby alleviating the progression of AS. However, the precise molecular mechanisms underlying *C. butyricum*-mediated regulation of linoleic acid metabolism remain to be fully elucidated. Potential modulators include butyrate secretion or other bioactive molecules produced by this species, though further research is needed to confirm their roles.

In addition, *C. butyricum* administration markedly attenuated HFD-induced body weight gain in *Apoe^−/−^* mice, suggesting its potential anti-obesity effects. Obesity-driven chronic low-grade inflammation in metabolically impactful tissues (e.g., adipose depots) has been mechanistically linked to atherogenesis [51,52,53]. Consequently, the attenuation of systemic inflammation observed following *C. butyricum* intervention may be partially due to its anti-obesity effects. This effect may be in part attributed to its ability to inhibit intestinal fat absorption, thereby reducing systemic lipid uptake and energy harvest from the HFD. Notably, emerging evidence has indicated that microbial metabolic activities may confound fecal fat quantification, including commensal *Fusimonas*-mediated fatty acid synthesis [54] and cholesterol metabolism driven by *Oscillibacter* [8]. To address these limitations, radiolabeled tracers have been successfully employed to evaluate the intestinal fat absorption [55,56,57], as exemplified by oral gavage of ^3^H-triolein emulsified in olive oil followed by serial plasma radioactivity quantification, enabling precise assessment of intestinal absorption kinetics [58]. Moreover, given that linoleic acid exacerbates adiposity by enhancing the abundance of ARA cascade-associated PUFA metabolites in obesogenic environments [59], our findings collectively suggest that *C. butyricum* may confer dual therapeutic benefits against both AS and obesity through coordinated modulation of linoleic acid metabolic pathways. Furthermore, the anti-obesity effects of *C. butyricum* may be synergistically amplified not only through metabolic modulation of linoleic acid but also potentially via reduced intestinal lipid absorption. Nevertheless, the causal relationship between *C. butyricum*-mediated metabolic reprogramming of linoleic acid, fat deposition, and atherosclerotic progression remains to be mechanistically dissected.

While our data demonstrate the anti-atherogenic effects of *C. butyricum* through linoleic acid metabolic regulation, the precise mechanistic interplay between strain-specific bacterial functions and host metabolic adaptations remains to be causally established. Central to this ambiguity is determining whether the observed suppression of inflammatory cascades arises primarily from *C. butyricum*-derived bioactive metabolites directly modulating linoleic acid metabolism, or alternatively, from their capacity to limit the intestinal bioavailability of linoleic acid. Significantly, a low-fiber diet likely deprives *C. butyricum* of substrates (e.g., resistant starch) necessary for butyrate synthesis. Critically, exogenous butyrate fails to alter body weight in *Apoe^−/−^* mice, suggesting that the anti-atherogenic effects of *C. butyricum* may depend on metabolites beyond butyrate [60]. Disentangling these possibilities necessitates systematic investigation, which could involve employing complementation studies with either *C. butyricum*-derived specific metabolites or other gut bacterial species, in combination with gene editing techniques. Beyond linoleic acid metabolism, *C. butyricum* has ameliorated HFD-induced gut microbiota dysbiosis in obese mice, as evidenced by the restoration of obesity-associated reductions in Bacteroidota abundance and attenuation of Proteobacteria expansion [61]. Notably, the restored abundance of Bacteroidota—a phylum containing multiple species with documented anti-inflammatory properties [11,62,63]—indicates a potential microbiota-mediated mechanism through which *C. butyricum* might inhibit AS progression by indirectly attenuating systemic inflammation.

Despite its recognition as a SCFA-producing probiotic with established benefits in enhancing intestinal barrier integrity [61], suppressing lipopolysaccharide (LPS)-mediated inflammation [64], and restoring gut microbiota homeostasis [21], *C. butyricum* has not yet achieved broad approval as a dietary probiotic for humans. This limited adoption may stem from insufficient mechanistic insights into its health-promoting effects in humans and unresolved safety concerns. Here, we reveal that *C. butyricum* significantly attenuates systemic inflammation and atherosclerotic plaque progression triggered by a high-fat diet. Mechanistically, these protective effects are mediated through selective modulation of host linoleic acid metabolism. Critically, the metabolic flexibility of linoleic acid pathways across mammalian systems suggests that this mechanism may retain translational relevance in humans, particularly given the conserved role of linoleic acid-derived mediators in vascular inflammation. Our findings not only elucidate a previously unrecognized molecular pathway underlying *C. butyricum*’ s bioactivity but also provide critical evidence to support its translational development as a next-generation probiotic (NGP) for the management of inflammation and metabolic disorders. To accelerate clinical implementation, future studies should prioritize human trials evaluating the dose-responsive efficacy of *C. butyricum* in suppressing pro-atherogenic linoleic acid metabolites in parallel with cardiovascular risk biomarkers, while accounting for inter-individual variations in baseline microbiota and dietary fat intake.

Linoleic acid has been associated with beneficial health effects, notably through epidemiological evidence demonstrating an inverse correlation between increased dietary intake and cardiovascular risk [65]. Its anti-inflammatory properties have been mediated through suppression of Th17 cell differentiation and enhancement of Treg cell development [66]. However, ARA and its metabolic derivatives derived from linoleic acid metabolism have been implicated in AS progression through promoting systemic inflammation and foam cell formation [32,67]. Under pathological conditions such as HFD-induced dyslipidemia, the linoleic acid metabolic pathway is pathologically activated, as evidenced by elevated levels of linoleic acid and its derivative ARA [32]. Notably, *C. butyricum* treatment significantly attenuated HFD-induced AS in *Apoe^−/−^* mice, primarily through suppression of linoleic acid-derived pro-inflammatory metabolites, including ARA-mediated mediators such as EETs, LTF4, and LTC4. Our data further underscore the pro-atherogenic role of dysregulated linoleic acid metabolism. These findings collectively demonstrate the critical importance of maintaining linoleic acid metabolic homeostasis as a strategic target for AS management.

## 5. Conclusions

In summary, this study uncovers novel mechanistic insights into the atheroprotective role of *C. butyricum*. We demonstrate that *C. butyricum* supplementation attenuates AS progression in HFD-fed *Apoe^−/−^* mice by suppressing systemic inflammation and improving dyslipidemia. Crucially, these benefits are mediated through modulation of linoleic acid metabolism, evidenced by reduced levels of linoleic acid-derived metabolites. Collectively, our findings establish a mechanistic framework for targeting the gut microbiota-host metabolic crosstalk, positioning *C. butyricum*-mediated modulation and linoleic acid metabolic homeostasis restoration as translational therapeutic strategies to mitigate AS.

## Data Availability

The original contributions presented in this study are included in the article/Supplementary Material. Further inquiries can be directed to the corresponding authors.

## Supplementary Materials

The following supporting information can be downloaded at: https://www.mdpi.com/article/10.3390/microorganisms13061220/s1, Figure S1: Compounds classification of the differential metabolic pathways between PBS and *C. butyricum*-treated groups in negative (up) and positive (down) ionization modes. Table S1: The KEGG pathway analysis of differential metabolites between PBS and *C. butyricum*-treated groups in negative ionization modes. Table S2: The KEGG pathway analysis of differential metabolites between PBS and *C. butyricum*-treated groups in positive ionization modes. Table S3: Specific primers for quantification of *C. butyricum*.

## Abbreviations

AS	Atherosclerosis
CB	*Clostridium butyricum*
ASCVD	Atherosclerotic cardiovascular disease
CVD	Cardiovascular disease
DSS	Dextran sodium sulfate
SCFAs	short-chain fatty acids
HFD	high-fat diet
Th17	T helper cell 17
Treg	Regulatory cell
PCA	Principal component analysis
PLS-DA	Partial least squares-discriminant analysis
CFU	Colony-forming units
TG	Triglycerides
LDL-C	Low-density lipoprotein cholesterol
HDL-C	High-density lipoprotein cholesterol
TC	Total cholesterol
qPCR	Quantitative PCR
PFA	Paraformaldehyde
OCT	optimal cutting temperature
LC-MS	Liquid chromatography-tandem mass spectrometry
KEGG	Kyoto encyclopedia of genes and genomes
PUFA	Polyunsaturated fatty acid
ARA	Arachidonic acid
COX	Cyclooxygenase
LOX	Lipoxygenase
CYP450	Cytochrome P450
EET	Epoxyeicosatrienoic acid
5, 6-DHET	5, 6-dihydroxyeicosatrienoic acid
LTF4	Leukotriene F4
LTC4	Leukotriene C4
8,15-diHETE	8,15-dihydroxyeicosatetraenoate
ox-LDL	Oxidized LDL
NGP	Next-generation probiotic
LPS	Lipopolysaccharide
