# Supplementary material for: Clostridium butyricum Ameliorates Atherosclerosis by Regulating Host Linoleic Acid Metabolism
Source: Microorganisms. 2025 May 27;13(6):1220. doi: 10.3390/microorganisms13061220 (PMC12194821; doi:10.3390/microorganisms13061220)
Supplement: Supplementary file 1 [file microorganisms-13-01220-s001.zip › microorganisms-3603302-supplementary.pdf]

microorganisms

MDPI

SUPPLEMENTARY MATERIALS

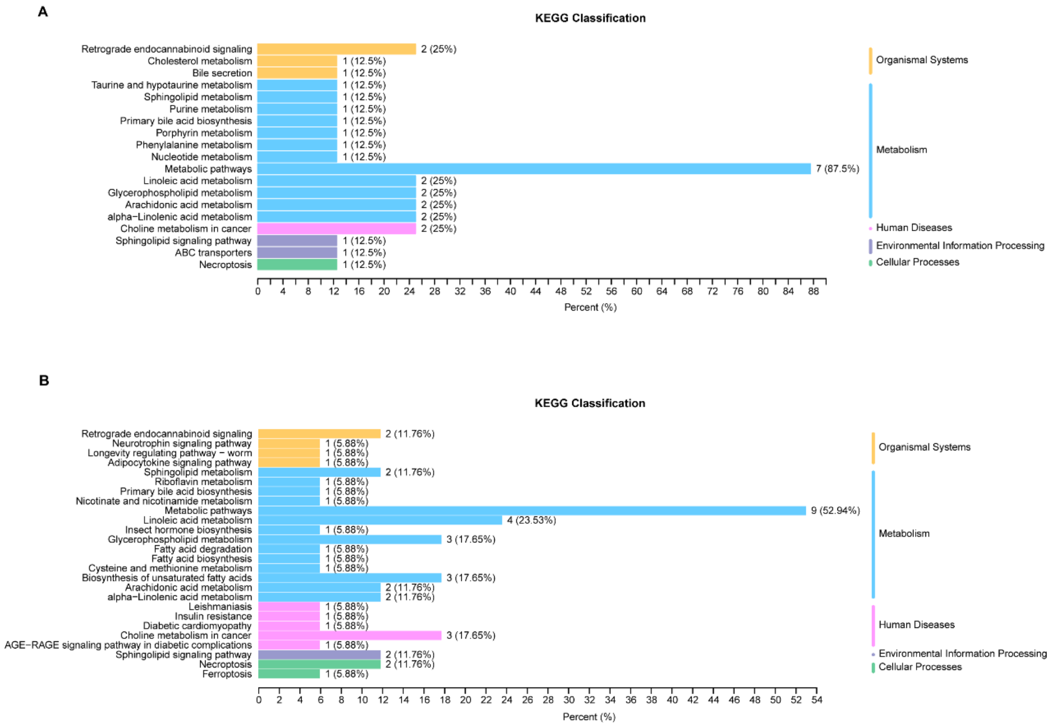

Figure S1. Compounds classification of the differential metabolic pathways between PBS and *C. butyricum*-treated groups in negative (up) and positive (down) ionization modes.

Table S1. The KEGG pathway analysis of differential metabolites between PBS and *C. butyricum*-treated groups in negative ionization modes.

KEGG_Pathway	*p*-Value	Compound Count	Rich Factor
alpha-Linolenic acid metabolism	0.03643	9	0.22222222
Linoleic acid metabolism	0.0447	10	0.2
Retrograde endocannabinoid signaling	0.05363	11	0.18181818
Choline metabolism in cancer	0.08391	14	0.14285714
Glycerophospholipid metabolism	0.09503	15	0.13333333
Cholesterol metabolism	0.10517	3	0.33333333
Necroptosis	0.10517	3	0.33333333
Taurine and hypotaurine metabolism	0.10517	3	0.33333333
Arachidonic acid metabolism	0.1066	16	0.125
Sphingolipid metabolism	0.16979	5	0.2

Table S2. The KEGG pathway analysis of differential metabolites between PBS and *C. butyricum*-treated groups in positive ionization modes.

KEGG_Pathway	*p*-Value	Compound Count	Rich Factor
Linoleic acid metabolism	0.00306	10	0.4
Necroptosis	0.00495	2	1
Biosynthesis of unsaturated fatty acids	0.00926	7	0.42857143
Sphingolipid signaling pathway	0.0623	6	0.33333333

AGE-RAGE signaling pathway in diabetic complications	0.07234	1	1
Adipocytokine signaling pathway	0.07234	1	1
Leishmaniasis	0.07234	1	1
Neurotrophin signaling pathway	0.07234	1	1
Retrograde endocannabinoid signaling	0.08351	7	0.28571429
Sphingolipid metabolism	0.10663	8	0.25

Table S3. Specific primers for quantification of *C. butyricum*.

**Primers**	**Sequence (5′-3′)**
Uni16s-F	ACTCCTACGGGAGGCAGCAGT
Uni16s-R	ATTACCGCGGCTGCTGGC
C. butyricum-F	TACCGCATGGTACAGCAATT
C. butyricum-R	TCGCGAGGTTGCATCTCAT
